# Chemical and palaeoentomological evidence of a relationship between early Eocene Belgian and Oise (France) ambers

**DOI:** 10.1038/s41598-024-64286-z

**Published:** 2024-06-14

**Authors:** Leyla J. Seyfullah, Jacek Szwedo, Alexander R. Schmidt, Cyrille Prestianni

**Affiliations:** 1 Department of Palaeontology, University of Vienna, Josef-Holaubek-Platz 2, 1090 Vienna, Austria; 2 Laboratory of Evolutionary Entomology and Museum of Amber Inclusions, Department of Invertebrate Zoology and Parasitology, University of Gdańsk, 59, Wita Stwosza Street, 80308 Gdańsk, Poland; 3 Department of Geobiology, University of Göttingen, Goldschmidtstraße 3, 37077 Göttingen, Germany; 4 OD Earth & History of Life, Royal Belgian Institute of Natural Sciences, Rue Vautier 29, 1000 Brussels, Belgium; 5 Evolution and Diversity Dynamics Laboratory (EDDy Lab), Geology Department, Liège University, Liège, Belgium

**Keywords:** Inclusion, Miridae, New genus, New species, New combination, Resin, Palaeontology, Taxonomy

## Abstract

Of the early Eocene amber deposits known across the world, Belgian amber has been mostly absent from the relevant literature. We reinvestigated amber held in the palaeobotanical collection of the Royal Belgian Institute of Natural Sciences, Brussels, which derived from three localities in Belgium that originated from two geographical areas (Leval-Trahegnies and Orp-le-Grand). Using Fourier transform infrared (FTIR) spectroscopy we show the close chemical relationship of Belgian amber to the early Eocene Oise amber from the Paris Basin, and highlight the potential effect of weathering on the amber chemistry. The amber derives from a very similar botanical source as the Oise amber (Combretaceae or Leguminosae-Caesalpinioideae), but from different coeval basins. The two Leval-Trahegnies localities provided amber that exhibit different stages of weathering (heavily fissured and crazed, darkened) and lacking any inclusions. The Orp-le-Grand locality provided the least weathered amber, with one amber piece containing two inclusions: a mite and a new genus and species of hemipteran (*Cativolcus uebruum* gen. et sp. nov.), and a second one that preserved the impression of insect wings pressed into the surface.

## Introduction

Various significant amber deposits have been reported from the Ypresian (early Eocene). These include Cambay amber from the Vastan and Tadkeshwar lignite mines in Gujarat, Western India, which is a fairly large 54.5 Ma deposit^1^ with diverse arthropod inclusions from a tropical ecosystem, and Dipterocarpaceae have been identified as the amber source plants^2^. Fushun amber from Liaoning, China has a highly diverse 50–53 Ma palaeobiota preserved in fossilized cupressaceous resin^3^. Insect inclusions from the Hat Creek amber deposit, British Colombia, Canada, were dated to the early Eocene Hat Creek Coal Formation^4^. In France, the Oise amber from Le Quesnoy in the Paris Basin is of high quality and is very fossiliferous with over 300 arthropod species recorded^5,6^. The Oise amber derives from the earliest Eocene of the Paris Basin, about 56 Ma, and the fossil plant *Aulacoxylon sparnacense* (Combretaceae or Leguminosae-Caesalpinioideae (Fabaceae)) is thought to be the likely source based on associated anatomically preserved woods^6,7^. These Ypresian-aged amber deposits occurred during a time of global warming marked by the Paleocene Eocene Thermal Maximum (PETM) and reaching a maximum at the Early Eocene Climatic Optimum (EECO)^8,9^.

The first Ypresian-aged Belgian amber inclusion, now considered lost, was described by Meunier^10^ and then forgotten. Later, Langeron^11^ described Ypresian amber from Leval, in Hainaut, mentioning the presence of (unprepared and unstudied) insect inclusions, then 55 years later a short summary was given^12^. Little else was recorded of these ambers except for the mention in grey literature of two insect inclusions in ambers from two different localities, thought to be those mentioned by Langeron^11^. These specimens have also since been lost and interest in the amber waned.

Here we describe historically collected Belgian amber from three localities in two geographical areas (Leval-Trahegnies and Orp-le-Grand) now held in the palaeobotanical collection of the Royal Belgian Institute of Natural Sciences, Brussels. The amber originating from the two Leval-Trahegnies localities was found to lack inclusions and to be very weathered. However, the Orp-le-Grand locality yielded previously unrecorded inclusions; a new genus and species of true bug, and a heavily pyritized mite. Additionally, on the surface of one stalactite-shaped amber piece, there are the impressions of insect wings preserved. The chemical analyses of these early Eocene Belgian ambers show their close relationship to each other and to the contemporaneous Oise amber from the Paris Basin, implying the same or a very similar source plant (Combretaceae or Leguminosae-Caesalpinioidceae) for all these different amber localities.

### Geological age and setting

**Figure 1:**
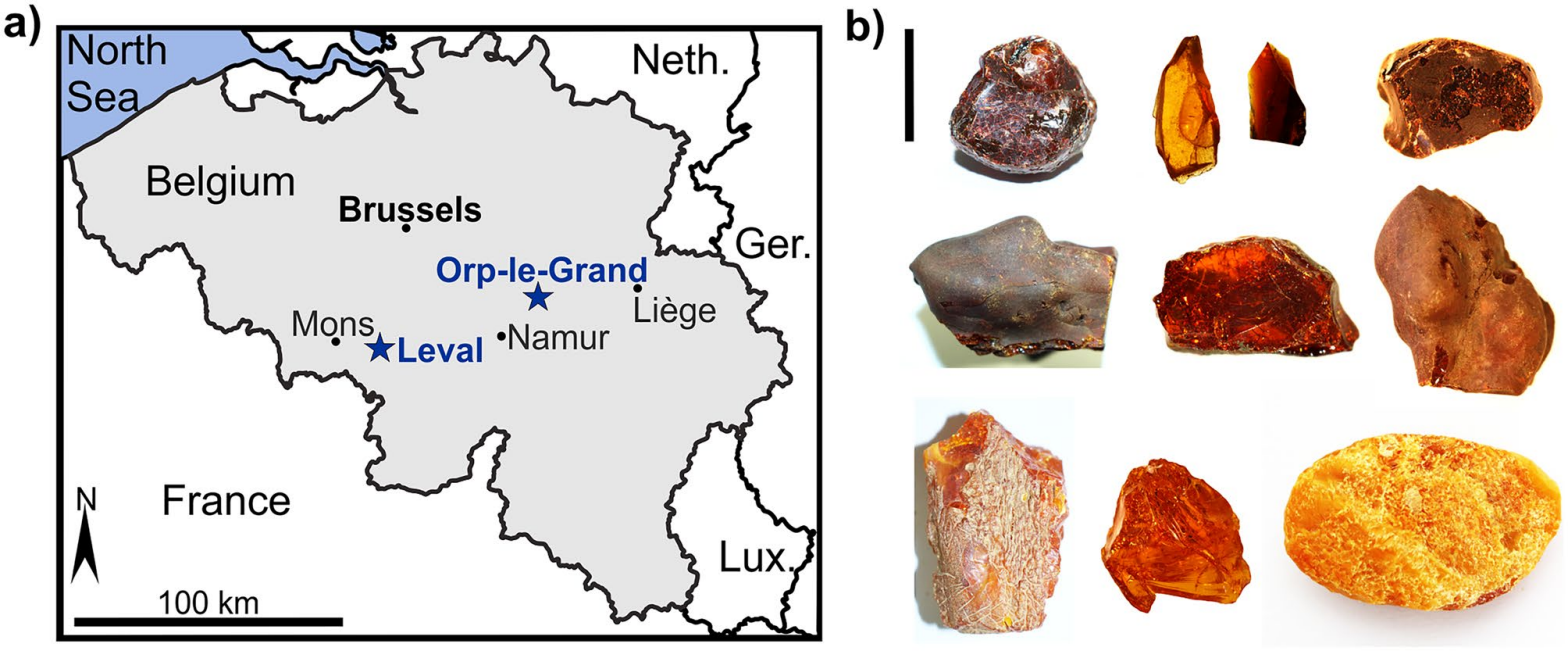
Ambers from Belgium. (**a**) map of Belgium and surrounding area, with the Eocene amber-bearing localities represented in the RBINS collections (starred). Ger.—Germany, Lux.—Luxembourg, Neth.—Netherlands. (**b**) Eocene ambers from Belgium, top row, amber from *Sablière La Courte à Leval* (M5), this is the most darkened and fissured amber from the three localities, with some variations in colour; middle Row, amber from *Trieu de Leval* (M6); lowest row, amber from *Orp-le-Grand* showing the lightest colour of amber present from each of the three localities. Scale bar 20 mm.

The ambers from the palaeobotanical collection in the Royal Belgian Institute of Natural Sciences (RBINS, Institut Royal des Sciences Naturelles de Belgique) derive from three amber deposits recovered from two areas: *Orp-le-Grand* and Leval-Trahegnies (one locality labelled as *Sablière La Courte à Leval* (M5), and the other as *Trieu de Leval* (M6)) (Fig. 1a starred). These deposits were listed in the collection as dated to the Landénien supérieur/upper Landenian, now an obsolete stratigraphic term, or as Sparnacien/Sparnacian (describing a facies). The Belgian ambers studied here have general inventory (IG, Inventaire Général) numbers registered in the collection catalogues of the RBINS, indicating their provenance. The amber labelled as *Sablière La Courte à Leval* (M5) has the number IG 6978 and that from *Trieu de Leval* (M6) the number IG 7021, while the *Orp-le-Grand* amber is identified with the number IG 9875.

In the Leval-Trahegnies area, the *Trieu de Leval* and *Sablière de La Courte* (correct name for the *Sablière La Courte à Leval*) localities are only separated by a few hundred meters. They however show slightly different successions. In the *Trieu de Leval* locality, first mentioned by Rutot^13^, a thick grey clay deposit is intercalated between chalk deposits of the Late Cretaceous Saint-Vaast Formation and green sands of the Ypresian Carnières Formation corresponding to the Orchie Member of the Kortrijck Formation (the first name has been used by cartographers^14^ and defined there but was not yet recognized by the National Commission for Stratigraphy Belgium (NCSB)). By contrast, in the *Sablière de La Courte* locality, the Saint-Vaast Formation is overlain by a thick layer of cross-bedded sands themselves covered by a layer of grey clay^18^. Both the sand and the clay layers have been eroded by quaternary fluvial deposits. Chauffin^12^ later stated that there are two superimposed levels (one Paleocene- and the other Eocene-aged) exposed in quarries in Leval-Trahegnies, but amber has only been reported from the Eocene level in this area^14^. Amber occurs in both localities in lignite beds intercalated within the layer of grey clay. Moreover, in the *Trieu de Leval* locality, the amber is associated with abundant plant remains including branches and leaves^14^. Originally, only one insect inclusion was mentioned from the *Sablière de La Courte* locality. It was determined by a local researcher, M. Séverin, as a “hémiptère homoptère de la famille des Jassides”, and reported by Rutot^14^.

In these early reports, the determination of the age and of the lithostratigraphy has been matter of debate. Rutot^13^ attributes the *Trieu de Leval* locality to the Montian, an obsolete continental regional stage dated from the early-middle Paleocene, while Briart^15^ reported it as the Late Cretaceous Heersian local stage. In an attempt to clarify this point, the above-mentioned leaf flora has been studied by Marty^16^ who concluded that the flora was younger than the Cretaceous and likely Paleocene. Due to the intercalation of sands, the *Sablière de La Courte* locality has been dated as upper Landenian in age^17^. This regional stage would now correspond to the lower Eocene (Ypresian). A better understanding of the geological succession in the region has allowed the determination that both deposits were part of the same geological formation. Older literature attributes both of the Leval localities to the Tienen Formation^17^, however, the most recent data places them within the contemporaneous Erquelinnes formation^18^. The latter formation has not yet been recognized by the NCSB but as it is used in the most recent publication^18^ we prefer to mention both names. This formation is unofficially divided in two members: the sandy La Courte Member and the Leval Member that is made of grey clays. It is within the Leval Member that the amber occurs. The depositional setting for both the Leval-Trahegnies and Orp-le-Grand geographical areas are interpreted as fluvio-lacustrine sediments rich in organic matter^19^ corresponding to a swampy environment^18,20^.

The amber from the Tuillerie of the *Orp-le-Grand* locality is first mentioned by Stockmans^21^ but, as far as we know, no detailed account was ever published on this locality. Despite the lack of stratigraphic description, the stratigraphic unit positioning of Defino & Smith (see Fig. 4 of Delfino & Smith)^19^ indicates that the amber deposit can be attributed to the upper part of the Tienen Formation.

The base of the Tienen Formation is marked by a carbon isotope excursion (CIE) marking the onset of the Paleocene–Eocene Thermal Maximum (PETM) and therefore recording the Paleocene–Eocene boundary^22,23^. The Tienen Formation has been studied in detail for its fossil content and has been determined as being early Ypresian, early Eocene in age^24,25^, with the Erquelinnes formation considered to have the same age^24^. As the ambers were washed clean before or at the time they came into the collections, there is no additional material such as the host sediment available for palynological dating.

## Results

### Appearance of ambers from Leval

No inclusions were observed in either the *La Courte à Leval* (M5) or the *Trieu de Leval* (M6) ambers. Visually the ambers are both very difficult to screen with light microscopy as they are now very crazed and darkened. The *La Courte à Leval* (M5) amber is predominantly dark red in colour, and the most crazed and fissured of all the ambers. There are a few yellow opaque pieces, but these constitute a very small proportion of the material. The amber is now mostly angular fragments and was likely mostly unlayered (Fig. 1b top row). The *Trieu de Leval* (M6) amber shows a mixture of colours from yellow, to orange and brown, with orange-red angular fragments being predominant, the yellow material is opaque. There is very little layered material. As the amber pieces are highly crazed and fissured, it is difficult to see inside the pieces to determine if they are translucent or not (Fig. 1b middle row).

### Appearance of amber from *Orp-le-Grand*

The amber has a more or less uniform high translucency and a medium orange colour, although there is 50 g of yellow and opaque (3.8% by weight) amber pieces present. The majority of the amber is unlayered, fissured and crazed, although this is the least fissured, darkened and crazed amber of the three amber-bearing localities investigated here. There are some stalactite-like and layered pieces in the collection (Fig. 1b lowest row). This is the only amber in the RBINS collections that yielded new fossil inclusions and impressions.

### FTIR-ATR analyses of Belgian ambers

**Figure 2:**
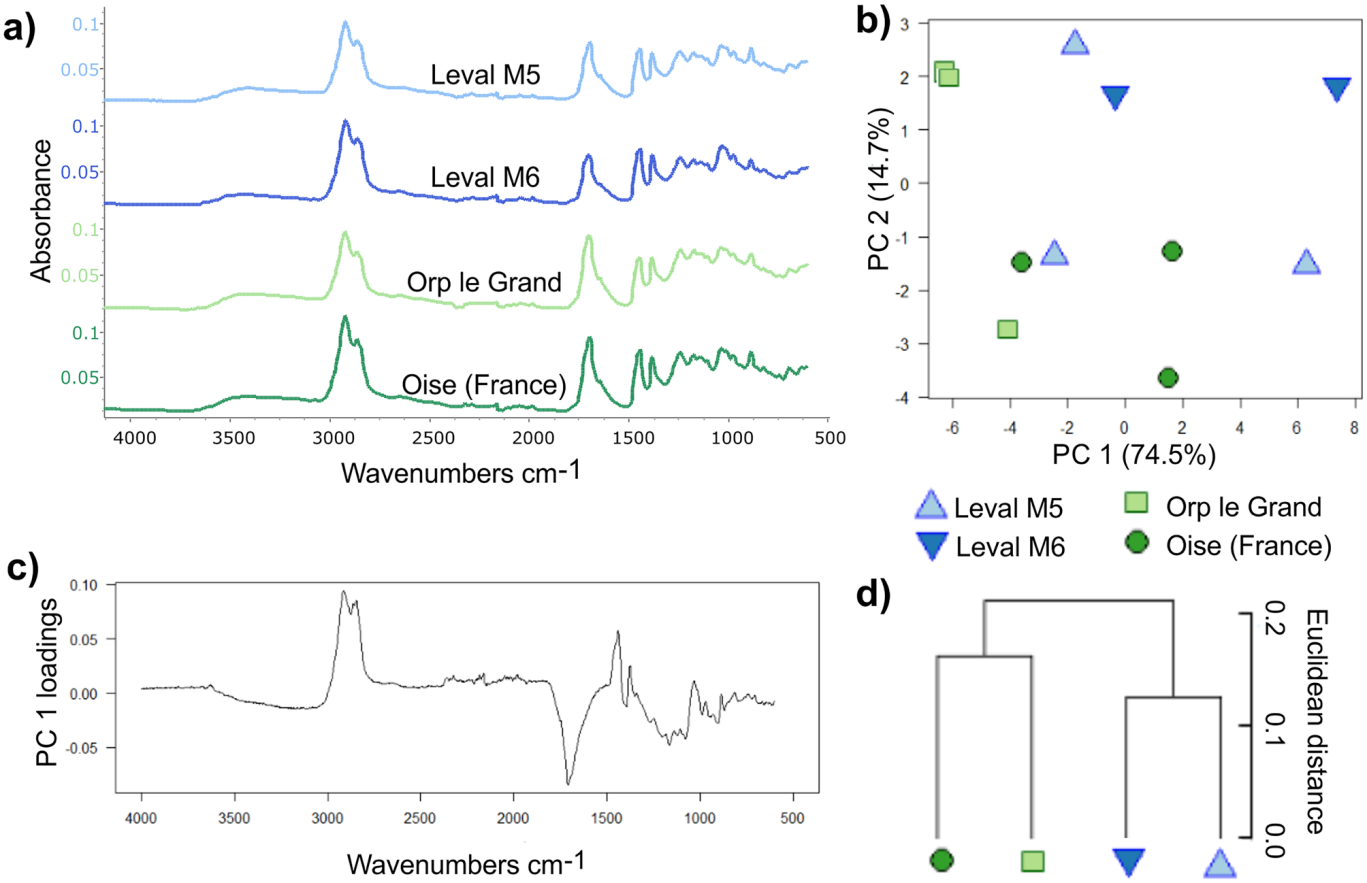
Analyses of Belgian and Oise ambers. (**a**) Mean FTIR spectra by locality for the Eocene ambers from Belgium (Leval M5 = *La Courte à Leval* (M5), Leval M6 = *Trieu de Leval* (M6), and *Orp-le-Grand*) and from Oise (France). (**b**)–(**d**) Multivariate analysis of amber spectra from Belgium Leval M5 = *La Courte à Leval* (M5), Leval M6 = *Trieu de Leval* (M6), and *Orp-le-Grand*) and from Oise (France) using FTIR-ATR spectroscopy: (**b**) Principal components analysis showing PC1 vs. PC2, note the two triangles for Leval M6 (= *Trieu de Leval* (M6) – the more central one is composed of two triangles, one superimposed on the other, giving the appearance that one sample is missing when in fact two are indistinguishable; (**c**) loadings for PC1; (**d**) mean cluster analysis by locality.

In order to understand the potential relationships between and infer any source plant(s) for the Belgian ambers, FTIR-ATR spectroscopy was used to analyze the bulk chemistry, and the results were directly compared with those spectra obtained from Oise (Le Quesnoy quarry) amber samples (Supplementary figure S1). All samples have the characteristic peaks that indicate that all the samples are fossil resins (Table 1, Fig. 2a).

**Table 1:** Diagnostic peaks for fossil resins, data compiled from Tappert et al.^26^.

Wavenumber cm^−1^	3400	3076	2935; 2848	2870	1693
Peak type	Wide peak	Small peak	Dominant peak, small peak	Small peak	Large peak
Cause	O–H bonds stretching	C–H stretching of monoalkyl groups	Methylene groups	Methyl groups	C–O double bonds in carboxyl groups of resin acids

The fingerprint region below 1500 cm^−1^ can vary greatly in its peaks depending on the amber or resin tested. Here all the ambers tested share peaks at 1447 cm^−1^ and 1384 cm^−1^ linked to C–H bending motions of methyl and methylene functional groups, the peaks at between 1300 and 1100 cm^−1^ are assigned to C–O single bonds and the peak at 887 cm^−1^ is due to the out-of-plane C–H bending motions in terminal methylene groups^26–29^. The mean FTIR spectra (Fig. 2a) and the individual spectra (Supplementary figure S1) for the different ambers are almost identical. Furthermore, the Oise and the Belgian ambers all share the same functional groups shown by the presence of the same peaks in their spectra (Table 2, Fig. 2a, Supplementary figure S1). There are small variations in the peak intensities observable (Supplementary figure S1) meaning that the spectra are not completely identical as the peaks vary in intensity, but they all share the same bulk chemistry.

**Table 2:** Peaks and functional group interpretation of the Belgian and Oise amber mean FTIR-ATR spectra, *v*, stretching; *as*, asymmetric; *s*, symmetric; *δ*, in plane bending. data compiled from Tappert et al.^26^ Beltran et al.^27^, Lyons et al.^28^, Pretsch et al.^29^.

Wavenumber cm^−1^	Peak type	Functional group	Belgian amber *Trieu de Leval* (M5)	Belgian amber *Trieu de Leval* (M6)	Belgian amber *Orp-le-Grand*	Oise amber
3400	Wide peak	*v*O–H bonds stretching of alcohols and/or carboxylic acids/*vs* OH	Present	Present	Present	Present
3078	Small peak	*vs*CHn deformation vibrations of =C–H groups/alkynes	Present	Present	Present	Present
2930	Dominant peak	*vas*CHn aliphatic stretching of methylene groups	Present	Present	Present	Present
2865	Small peak	*v*CHn aliphatic stretching of methyl groups	Present	Present	Present	Present
2848	Small peak	*vas*CHn aliphatic stretching of methylene groups	Present	Present	Present	Present
1722	Minor peak or slope	*v*C=O double bonds	Present	Present	Present	Present
1693	Large peak	*v*C=O double bonds in carboxyl groups of resin acids	Present	Present	Present	Present
1644	Minor peak	*δ*O–H bending bond or exomethylene, or unsaturated *v*C–C bond	Present	Present	Present	Present
1447	Peak	*δas*C–H bending motions of methyl and methylene groups	Present	Present	Present	Present
1384	Peak	*δs*C–H vibrations of methyl and methylene groups	Present	Present	Present	Present
1242	Peak	*vas*C–O single bond stretching	Present	Present	Present	Present
1150	Tiny peak on shoulder	*vas*C–O single bond stretching	Present	Present	Present	Present
1005	Small peak	*as*C–O–O or *v* CO–OH	Present	Present	Present	Present
1034	Peak	*vas*C–O single bond stretching	Present	Present	Present	Present
976	Peak	*δ*C–H vibrations	Present	Present	Present	Present
887	Peak	=C–H out-of-plane bending motions in terminal methylene groups	Present	Present	Present	Present

### Statistical analyses of the amber spectra

In the multivariate analyses applied to the dataset (both the dataset and R scripting available in the Supplementary Dataset), 89.2% of the variance was accounted for by two principal components (percentage of variance for PC1: 74.52%, PC2: 14.65%, standard deviation for PC1: 5.03, PC2: 2.23), so no further principal components were needed to describe the variation. A baseline correction did not improve the results and was not needed as no baseline drift was observed.

The PCA (Fig. 2b) shows that the ambers from Leval (Leval (M5) and Leval (M6)) group together, whereas the *Orp-le-Grand* ambers group separately. The French Oise ambers group together and appear to overlap with a Leval (M5) sample. The loadings for PC1 (Fig. 2c) highlight the spectral regions of similarity and differences between the ambers. Firstly, the ambers share a similar level of resin polymerization, which is noted in the lack of peak intensity around 3400 cm^−1^ attributed to hydroxyl groups that are used to indicate degrees of resin polymerisation^18^. The intensity of the peaks at 2935 cm^−1^ and 2848 cm^−1^, linked to methylene groups and the large (negative) peak at 1693 cm^−1^ caused by C–O double bonds in carboxyl groups of resin acids just highlight the band intensity differences between the samples at those wavenumbers. These differences are most likely attributable to either the slightly different taphonomy of the ambers, or their subsequent geological history and varying levels of weathering/oxidation, as noted in the different colourations and levels of oxidised appearance of the ambers from the different localities, or a combination of both.

The locality mean cluster analysis (Fig. 2d) highlights the grouping of the ambers from Leval, Belgium as they form one cluster, with amber from *Orp-le-Grand* forming a second cluster with Oise amber from France. This appears to mirror the colouration and level of oxidation noted for the ambers from each locality, with the ambers from Leval being the most darkened and oxidized (heavily fissured and cracking), whereas the ambers from *Orp-le-Grand* (Fig. 1b) and Oise are generally lighter and less oxidized in appearance. Additional amber spectra were included to broaden the coverage of the dataset and the results remain very similar, supporting the initial inferences (Supplementary figure S2). Further cluster analyses (Supplementary figure S3) treating each sample spectrum individually has two main groupings recovered in all cases for the Belgian and Oise ambers. There is a consistent separate grouping of the Leval M5_2, Leval M6_1 and Leval M6_3 spectra, suggesting that these ambers from the two Leval localities are not as distinct as represented by the mean cluster analysis shown in Fig. 2d. This could be because these three amber samples looked more alike in terms of colouration and crazing than the other ambers tested.

### Inclusions and impression in amber

**Figure 3:**
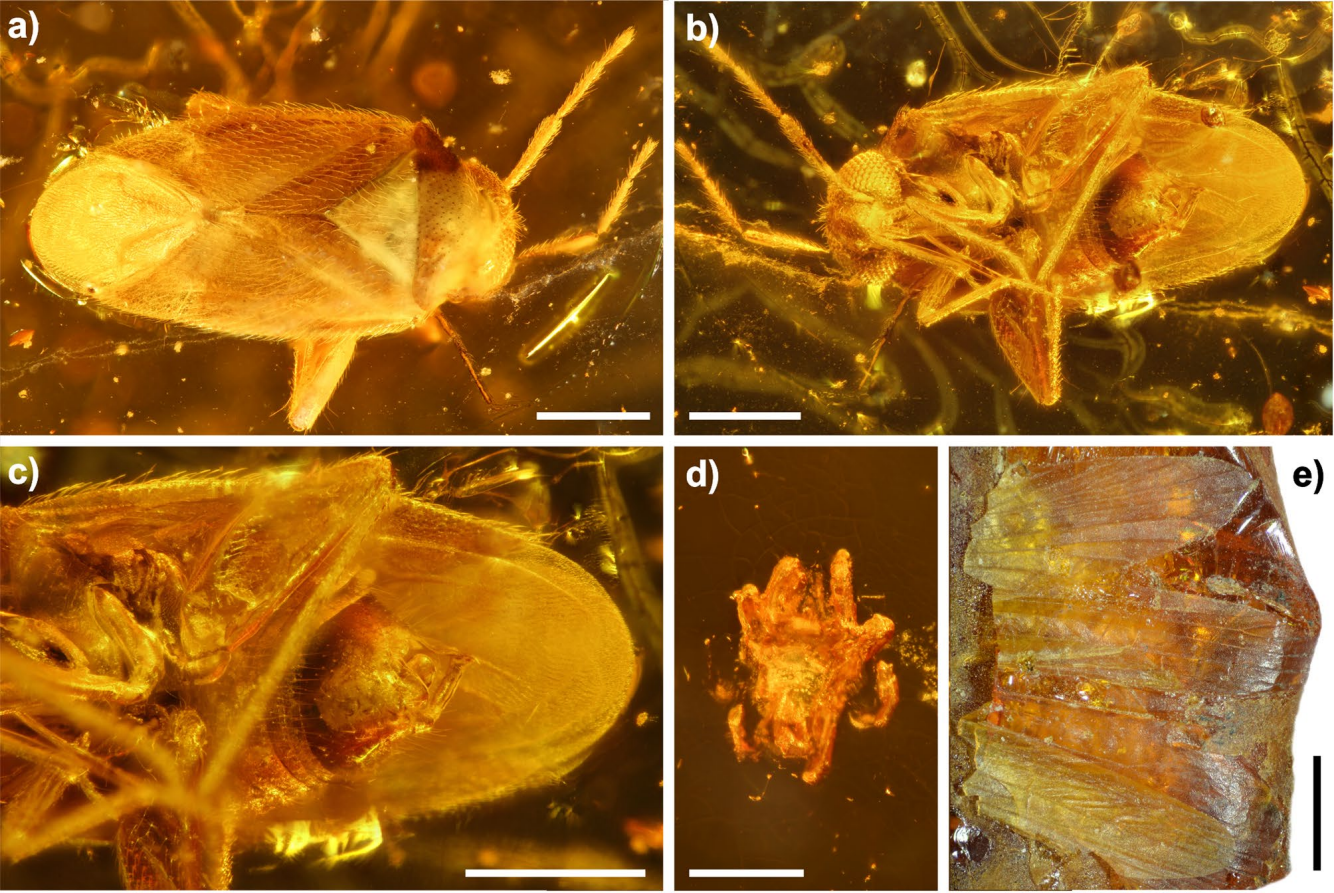
Arthropod fossils (inclusions and impression) in Belgian amber. (**a**)**–**(**c**) *Cativolcus uebruum* gen. et sp. nov. (BE-RBINS-ENT-AMBER-IG-34605). (**a**) dorsal side (40 stacked images), (**b**) ventral side (67 stacked images), (**c**) detail of genitalia (45 stacked images), (**d**) remains of degraded mite syninclusion, (**e**) impression of insect wings on amber surface. Scale bars 500 µm.

The only amber inclusions found to date are from one amber piece derived from the *Orp-le-Grand* locality (Fig. 3a–d). In this amber piece (BE-RBINS-ENT-AMBER-IG-34605) two inclusions were discovered. The larger (2.5 mm long) and better-preserved inclusion is of a hemipteran *Cativolcus uebruum* gen. et sp. nov. (Fig. 3a–c). The syninclusion is a mite (Fig. 3d), approximately 1.2 mm long but the development of pyrite has obscured most of the details of the specimen, preventing further identification. In addition to these inclusions, the impression of insect wings is preserved pressed into the surface of a separate stalactite-shaped amber piece (Fig. 3e).

## Discussion

### Comparison of Belgian ambers with Oise amber

FTIR analyses of Oise and Belgian amber samples showed that overall, the spectra from all localities are very similar (Fig. 2a, Supplementary Fig. 1), whereas the PCA (Fig. 2b) highlights that both sets of samples from Leval group together as they share a very similar origination and geological history. The *Orp-le-Grand* amber is more distinct, perhaps indicating a slightly different depositional environment and/or geological history (Fig. 2b, c). It also strongly suggests that there is an effect from degrees of weathering/oxidation being detected. The less heavily weathered *Orp-le-Grand* amber is more distant (Fig. 2d) to both more heavily weathered and darkened samples from Leval (compare colouration in Fig. 2b). The overall lighter, less crazed and fossiliferous amber from *Orp-le-Grand* clusters with Oise amber (Fig. 2d) suggesting they have the most similarity in bulk chemistry. In any case, this hints at the potential source plant being similar or even identical and similar environmental settings. As the samples from *Orp-le-Grand* are the least weathered of the Belgian ambers and the Oise sample appeared unweathered at the time of measurement, the level of weathering/oxidation in the Leval samples may account for their greater distance from the other samples (Fig. 2d). It is not likely that the apparent similarity between the *Orp-le-Grand* and Oise ambers results from a very closely shared geological history given that the Leval ambers, not the amber from *Orp-le-Grand*, are actually palaeogeographically closer to the Oise amber from the Paris Basin in France (Fig. 1).

Oise amber from France was first described by Lacroix^30^, and is thought to derive from the tree *Aulacoxylon sparnacense* (Combretaceae or Leguminosae-Caesalpinioideae) based on associated anatomically preserved woods^6,7^. Biomarker analysis of the amber identified the diterpene quesnoin, suggesting an affinity of the resin with Caesalpinioideae^31^. Nohra et al.^32^ confirmed an angiosperm amber source plant with likely fabacean affinity for Oise amber. Given the similarity of the FTIR spectra of Belgian and Oise ambers, we suggest that the Belgian ambers most likely have a similar or even identical Leguminosae-Caesalpinioideae botanical source (Fig. 2, Supplementary figure S1).

In addition to the ambers, notable similarity exists in the contemporaneous vertebrate faunas reported from localities in Belgium that are very close to the Belgian amber localities^5,33–35^. Dormaal, near Orp-le-Grand, is the reference-level MP7, the earliest European locality from the early Eocene^23,33–35^ and is correlated with Erquelinnes (near Leval-Trahegnies) and Le Quesnoy in France from which Oise amber derives. This adds independent evidence that these Belgian and French localities, and their ambers, were of comparable age and with similar environments and ecosystems.

### Inclusions in Belgian amber

Despite the very small amount of amber held in the palaeobotanical collections of the Royal Belgian Institute of Natural Sciences (7.9 kg from Leval in total and 1.3 kg from *Orp-le-Grand*), two inclusions in one amber piece and insect wing impressions on another were discovered from the *Orp-le-Grand* locality. The fissured and crazed surfaces of the Leval amber made it hard to be certain that there were no inclusions, but we could not discern any. We exclude the possibility that the newly discovered inclusions are the missing ones previously reported by Langeron^11^, as those historic specimens are reported from amber deriving from Leval, not *Orp-le-Grand*. We do not know the whereabouts of the insect inclusions from Leval reported by Langeron^11^.

The degradation of the mite inclusion through pyrite decay means that a closer identification is not possible. However, the new hemipteran is not affected by any pyrite, despite being contained in the same amber piece and actually shows excellent preservation. It is unclear why the two inclusions are preserved so differently given that the hemipteran had a clear fissure over its dorsal surface leading down towards the inclusion, but none were noted for the mite. The discovery of a new hemipteran genus and species is unexpected given the small amount of the Belgian ambers present in the palaeobotanical collection and that these ambers are almost completely barren of inclusions. This is particularly notable when compared to the richly fossiliferous and far larger, contemporaneous but completely separate Oise amber deposit from the Paris basin with 350 kg^5^, where Hemiptera comprise 10% of the currently known inclusions in Oise amber^36^.

#### Taxonomic treatment of hemipteran inclusion

Order: Hemiptera Linnaeus, 1758.

Suborder Heteroptera Latreille, 1810.

Infraorder Cimicomorpha Leston, Pendergrast & Southwood, 1954.

Superfamily: Miroidea Hahn, 1831.

Family: Miridae Hahn, 1831.

Subfamily: Psallopinae Schuh, 1976.

#### Genus: *Cativolcus* Szwedo gen. nov.

LSID urn:lsid:zoobank.org:act:005BA54B-E6AA-486E-8775-B1176305890D

Type species: *Cativolcus uebruum* Szwedo sp. nov.; here designated.

Composition. Type species *Cativolcus uebruum* Szwedo **sp. nov.** and *Cativolcus prokopi* (Vernoux, Garrouste et Nel^37^): Szwedo, comb. nov.

Etymology. Generic name is given after Cativolcus, a king of the Eburones, leader of a Belgic tribe of north-eastern Gaul (Gallia Belgica), where modern Belgium is located. Gender: masculine.

Holotype: (Fig. 3a–c); Specimen number BE-RBINS-ENT-AMBER-IG-34605, Ypresian, lower Eocene; Orp-le-Grand, Hainaut, Belgium.

### Diagnosis

It differs from *Isometopsallops* Herczek et Popov, 1992^38^, *Cylapopsallops* Popov et Herczek, 2006^39^ by smaller size, ca. 2.5 mm (about 4.8 mm in *Isometopsallops*, 5 mm in *Cylapopsallops*) length/width ratio of the head ca.2.2–2.4 (head distinctly longer, ca. 1.5 in *Isometopsallops*; ca. 1.6 in *Cylapopsallops*), anterior margin of vertex in contiguous with outline of compound eyes (vertex slightly protruded in *Isometopsallops*; vertex protruding in *Cylapopsallops* and in *Epigonopsallops* Herczek et Popov, 2009^40^); antennal segments of different length, with second antennomere the longest (subequal in *Isometopsallops*; second antennomere the longest in *Cylapopsallops* and in *Epigonopsallops*); rostrum exceeding end of metacoxae, but not reaching half of abdomen length (reaching terminalia in *Isometopsallops*; reaching middle of abdomen in *Cylapopsallops* and *Epigonomiris* Herczek et Popov, 1998^41^); metafemora widened and flattened (metafemora flattened in *Isometopsallops*; slender in *Cylapopsallops*; incrassate in *Epigonopsallops*).

### Description

Macropterous. Body length about 2.5 mm; body oblong oval, with margins parallel. Vestiture: surface generally smooth, dorsal surface of head, pronotum, mesonotum and tegmina covered with moderately short, erected and sub-erected hairs, directed posteriad; hairs on pronotum and corium lighter than background. Head wide, transverse, about 2.4 times as long as wide; jugae carinate. Vertex slightly convex, narrow, frons narrow, clypeus narrow. Combined length of antennomeres II and IV merely exceeding length of antennomere II. Pronotum in dorsal view subtriangular, anterior margin arcuate, inserted between compound eyes, posterolateral angles widely angulate, posterior margin shallowly concave; disc of pronotum smooth, anterior portion without distinct calli. Scutellum (mesonotum) wider at base than long. Hemelytrae flattened, commisura clavale about as long as mesocutellum. Membrane delicately crumpled, with two cells. Metafemora enlarged, flattened. Metatibia covered with dense, adpressed setae. Tarsi pseudo-trimerous (border between mid- and apical tarsomeres not distinct). Claws weakly curved, without subapical tooth. Male terminalia asymmetrical, right paramere enlarged, distinctly larger than left paramere.

Species: *Cativolcus uebruum*, Szwedo **sp. nov.**

(Fig. 3a–c; Supplementary figs. S4, S5).

LSID urn:lsid:zoobank.org:act:2D090065-1895-476A-A0E1-05424564DE7B

Etymology. Specific epithet from Gallic stem ‘uebru-’ – meaning amber.

### Holotype

Male. Specimen BE-RBINS-ENT-AMBER-IG-34605, deposited in the Royal Belgian Institute of Natural Sciences, Brussels, Belgium. Syninclusion: an undetermined mite.

### Diagnosis

Slightly smaller (2.12 mm long) than *Cativolcus prokopi* (2.25–2.5 mm); compound eyes with short setae between ocelli (compound eyes bare in *C. prokopi*); vertex, frons and clypeus with protruding setae (no such setae on head of *C. prokopi*); pronotum length/width ratio ca. 2.75 (ca. 2.36 in *C. prokopi*); ratio of corium to cuneus length ca. 2.92 (ratio 3.3–3.5 in *C. prokopi*); metafemur length/width ratio 3 (3.4 in *C. prokopi*).

### Description

Measurements. Total length (excl. antennae) 2.12 mm, maximum width 1.023 mm, length of body, 1.63 mm; length of antenna 1.06 mm. Body oblong oval. General colouration light brown to brownish. pronotum brownish with dark brown punctuation at bases of setae, scutellum (mesonotum) light brown; clavus, corium and cuneus brown to brownish. Dorsal surface of head, pronotum, mesonotum and tegmina covered with moderately short, erected and sub-erected hairs, directed posteriad; hairs on pronotum and corium lighter than background.

Head with compound eyes 0.56 mm wide, narrower than pronotum about 2/3 of its width. Head with compound eyes about 2.6 times wide as long (0.225 mm). Eyes large, discoid, occupying nearly entire sides of head, contiguous with anterior margin of pronotum and with anterior margin of vertex. Vertex narrow (0.09 mm long, 0.14 mm wide at base, 0.09 at apex), about half as width of compound eye, covered with moderately long, protruding setae. Frons and clypeus narrow, covered with moderately long, protruding setae. Clypeus smoothly flush with convex frons, about twice as long as wide; base of clypeus located slightly above than half eye height; mandibular plate (jugae) carinate, relatively broad, nearly reaching apex of clypeus. Ocelli absent. Antennal fossa situated at base of maxillary plate, emarginated, at about lower 1/3 of compound eye. Antenna 4-segmented, I antennomere 0.125 mm long, calyculate, narrow at base, slightly widening apicad; antennomere II the longest, 0.453 mm, about as wide as I antennomere, covered with dense, slightly protruding setae, longer than diameter of antennomere; III antennomere 0.281 mm long, narrower than II antennomere, covered with dense slightly protruding setae, longer than diameter of antennomere, apical antennomere, the IV, shorter than preapical one, 0.193 mm, narrowed at base, cigar-shaped, widening than tapering apicad, covered with slightly protruding setae, longer than diameter of antennomere, with single thicker seta in basal half. Rostrum 4-segmented, slightly exceeding apices of metacoxae; apical segment subequal to preapical one (ca. 0.24 mm).

Pronotum in dorsal view subtriangular, without calli, anterior margin widely arcuate, lateral margins distinctly diverging posteriad, posterolateral angles widely angulate, posterior margin shallowly concave. Pronotum about 2.8 times as wide (0.82 mm) as long in mid line (0.29 mm); about 1.23 times as long as head in total. Disc of pronotum convex, smooth, covered with moderately short, erected and sub-erected hairs, directed posteriad.

Scutellum (mesonotum) triangular, wider at base (0.60 mm) than long in mid line (0.39 mm). Scutellum lighter than pronotum and corium. Disc of scutellum flattened, smooth, covered with moderately short, erected and sub-erected hairs, directed posteriad.

Mesopleura with a few short setae in upper portion. Metapleura smooth, with scent gland area (?) delicately granulose and slit-like opening.

Procoxa and mesocoxa subequal, ca. 0.315 mm long, subconical, metacoxa slightly longer, ca. 0.355 mm. Profemur slightly flattened, angular, ca. 0.375 mm long; protibia slender, covered with short, adpressed setae, ca. 0.437 mm long. Protarsus pseudo-trimerous, ca. 0.18 mm long, basiprotarsomere 0.06, mid-and apical tarsomeres merely separated, subequal, ca. 0.155 mm long. Claws distinct, widely spread, not curved, without subapical tooth, parempodia short, setose (?). Mesofemur slightly longer than profemur ca. 0.437 mm, slightly flattened, angular, ca. 0.563 mm long. Mesotarsus same as protarsus.

Metafemur enlarged, flattened, ca. 0.76 mm long, 0.26 mm wide, covered with short setae; three longer, subapical setae at anteriad side, ventral margins with a few stronger setae. Metatibia narrow, ca. 0.99 mm long, covered with short, adpressed setae, about as long as metatibia diameter, four thicker and longer setae at apex. Metatarsus longer than pro- and mesotarsus, ca. 0.244 mm, pseudo-trimerous, basimetatarsomere shortest, ca. 0.06 mm, midmetatarsomere slightly shorter, ca. 0.09 mm than apical metatarsomere, 0.125 mm; tarsomeres covered with setae, tarsal claws distinct, widely spread, not curved, without subapical tooth, parempodia short, setose (?).

Hemelytra 1.74 mm long, 0.53 mm wide, corium 1.04 mm long, about 2.84 times as long as cuneus (0.36 mm), brownish, covered with moderately short, directed posteriad and lighter than background setae. Claval commisure 0.4 mm long. Hemelytral membrane with two cells, one small and one large, surface delicately crumpled; membrane 1.59 times as long as wide.

Male terminalia. Genital capsule (pygophore) entire, trapeziform in outline, smoothly rounded towards apex, covered with long setae; ventral margin with subtriangular process medially. Left paramere smaller than right one, elongate and rounded apically. Right paramere large, bent at base, with apical spine-like processes.

Age and occurrence. Ypresian, early Eocene; Orp-le-Grand locality, Belgium.

### Remarks

Psallopinae is the smallest subfamily within the true bugs family Miridae, including two extant genera – *Psallops* Usinger, 1946^42^ with 19 extant and 3 extinct species and *Isometocoris* Carvalho et Sailer, 1954^43^ with 2 recent species. The latter genus was proposed to be moved to Cylapinae, and Psallopinae reduced to tribe in Cylapinae^44^, but this action is not universally accepted^45,46^. In addition, extinct genera were described (*Cylapopsallops* Popov et Herczek, 2006^39^, *Epigonomiris* Herczek et Popov, 1998^41^, *Epigonopsallops* Herczek et Popov, 2009^38^, *Isometopsallops* Herczek et Popov, 1992^38^) and here established *Cativolcus* gen. nov., with *C. uebruum* **sp. nov**. and *C. prokopi* Vernoux, Garouste et Nel, 2010^37^ comb. nov. Distribution of Psallopinae covers the Old World tropics, subtropics and warm temperate regions: Saudi Arabia, Nigeria, Ghana, Congo, South Africa, Japan, Singapore, China, Taiwan and Thailand, Micronesia (Guam, Marian and Caroline Islands), and Australia^44,46^. Fossil taxa are reported from the lowermost Eocene amber of Oise^36^, Eocene Baltic amber (*Psallops eocenicus* Herczek, Popov et Gorczyca, 2015^44^, *Cylapopsallops kerzhneri* Popov et Herczek, 2006^39^, *Epigonomiris skalskii* Herczek et Popov 1998^41^, *Epigonopsallops groehni* Herczek et Popov, 2009^40^, *Isometopsallops schuhi* Herczek et Popov, 1992)^38,39,45^ and Bitterfeld amber (*Psallops bitterfeldi* Herczek, Popov et Gorczyca, 2015^45^), and Miocene Dominican amber (*Psallops popovi* Herczek, 2011^47^). *Cativolcus prokopi* (Vernoux, Garouste et Nel, 2010^37^) **comb. nov.** was originally described in the genus *Isometopsallops* Herczek et Popov, 1992^38^, but its distinctly smaller size, head length/width ratio ca. 2.2, different pronotum length width ratio ca.2.3 (*vs*. ca. 1.5 in *Isometopsallops*), tarsal claws without subapical tooth, exclude it from *Isometopsallops* and group in *Cativolcus* gen. nov. Bionomical data of the representative of the subfamily are very scarce^48^, habitats and habits are also poorly known, some species were collected at light, others by Malaise traps and net sweeping, with suggestion of their nocturnal habits. Some psallopinous bugs from Thailand have been found under half-detached bark fragments of fabaceous broad-leaf plants^49^. The psallopines are assumed to be also predaceous, similarly to related plant bugs of subfamily Isometopinae, which are also considered to be bark-inhabitants, as they are also documented under detached bark fragments, and prey on scale insects or other tiny arthropods^50,51^. Psallopinae is most probably a relict group that is closely related to the subfamily Isometopinae or Cylapinae^44,46,52–54^. Isometopinae, Psallopinae and Cylapinae should constitute a single clade, and these subfamilies are considered to be the basal (‘primitive’) groups among the other plant bugs Miridae^55,54,56^. Findings of fossil of Psallopinae in the amber of Oise and in the amber of Orp-le-Grand, Belgium, are for the moment the oldest fossil records of the group. *Cativolcus uebruum* **gen. et sp. nov.** from Belgian amber presents combination of such characters as enlarged eyes covering the greater part of the head, antennae placed at the inner sides of the eyes, reduced ocelli, the trapezoid pronotum, 2-segmented tarsi, placing it in Psallopinae. Recent Psallopinae are recognized by eyes occupying most of the head, the narrow vertex, the smooth body surface, the finely upturned anterior margin of the pronotum, subdivision of labial segment 4, the subapical tooth on the claws, tarsi 2-segmented, and membrane with single or two cells^46^. Large compound eyes are present in both species of *Cativolcus* gen. nov., such conformation of compound eyes is to be considered as the main apomorphic character supporting the clade Psallopinae + Isometopinae. The occurrence of ocelli is regarded as the plesiomorphic condition^57^, these are absent in *Cativolcus* gen. nov, therefore it could be derived condition of this fossil. Schuh & Schwartz^52^ suggested that loss of the ocelli in Psallopinae and Cylapinae could be convergent. Among the Miridae, presence of three-segmented tarsi is considered as the plesiomorphic condition^46,52,58^, however pseudotrimerous and dimerous tarsi are not exceptional in this family. Thus, this character is at least homoplasic^37^. With the present state of knowledge, it is hard to assess the phylogenetic value of morphological characters usually used as diagnostic for Psallopinae and its taxa. The most recent molecular phylogenetic results presented by Oh et al.^54^, suggested splitting of Isometopinae and Cylapinae in the earliest Cretaceous, alas Psallopinae were not taken to the analysis. The recent Psallopsinae live under very different climates even though all are warm, therefore it could be inferred that *Cativolcus uebruum* gen. et sp. nov., also inhabited a warm climate, which is in concordance for the palaeoconditions reconstructed for the fossil site and was present at a time of global warming when the resin was originally exuded entrapping the specimen.

## Conclusions

Early Eocene amber held in the palaeobotanical collection of the RBINS, Brussels, is from three localities across two geographical areas of Belgium. Using FTIR-ATR we showed these ambers to be chemically very similar to each other and to the contemporaneous Oise amber from France, suggesting that they share the same or a very similar botanical source (Combretaceae or Leguminosae-Caesalpinioideae). Ambers from the two different deposits of Leval-Trahegnies are more weathered in appearance (darkened and heavily crazed surfaces of the amber pieces) and they lack inclusions. The amber from *Orp-le-Grand* is, in comparison, less weathered as it is generally lighter in colour and less crazed in appearance than the Leval-Trahegnies derived amber. Although a smaller volume of amber is present from *Orp-le-Grand*, two inclusions were discovered in one piece, plus the impression of insect wings on the surface of another. The inclusions are a mite and a new genus and species of hemipteran (Psallopinae) *Cativolcus uebruum*, representing the shared oldest occurrence of the Psallopinae with that reported from the relatively palaeogeographically close by Oise amber from the Paris basin.

## Methods

### Material and historic localities

In the palaeobotanical collection of the Royal Belgian Institute of Natural Sciences (RBINS, Institut Royal des Sciences Naturelles de Belgique), Brussels, Belgium, there is amber collected from three localities which appear in the inventory as (1) *Sablière La Courte à Leval* (M5), (2) *Trieu de Leval* (M6), both from the Mons Basin, Leval-Trahegnies, near Morlanwelz in Hainaut Province, and (3) *Orp-le-Grand*, in the Walloon Brabant Province (Fig. 1 starred). The former amber-bearing localities have since been closed and filled in and thus cannot currently be accessed.

### La Courte à Leval (M5)

Notes from the RBINS collections record the M5 locality as ‘La Courte à Leval, Morlanwelz 5, Sablière la Courte à Leval-Trahegnies. Landénien Supérieurʼ. In the present analyses it is referred to as ‘Leval M5’. This amber was found in clay lenses in the sand quarry at La Courte à Leval. The whole deposit in the museum is marked with RBINS number Plateau PBot 6754 (67,939) and IG 6978. There is 4902 g of amber that has been washed and graded by size.

### Trieu de Leval (M6)

The M6 locality is listed as ‘Trieu de Leval, Morlanwelz 6 Carrières d’argile plastique du Trieu de Leval à Leval-Trahegnies, Landénien Supérieurʼ. In the present analyses it is referred to as ‘Leval M6’. The whole deposit in the museum is marked with RBINS number Plateau Pbot 6807 (67,903) and IG 7021. 2988 g of amber in total was recovered from a clay quarry, and has been washed and sorted by size.

### Orp-le-Grand

This amber locality is noted as ‘Orp-le-Grand, Argilière des Tuileries, Jauche 1, Landénien Supérieurʼ. In the present analyses it is referred to as ‘Orp le Grand’. The whole deposit from the Walloon Brabant Province in the museum is marked with RBINS number Plateau PBot 6808 and IG 9875. There is 1309 g of amber in total, which has been washed, sorted and selected by the seller of the amber who was the foreman of the quarry. The amber from this locality was sold to the museum in three separate lots (sometimes with other fossil materials). The IG number for Orp-le-Grand amber shows that it corresponds to the purchase on December 2, 1932 of mammal, reptile and fish bones as well as fossil resin from the brick and tile factory of Orp-le-Grand (pers. comms. Thierry Smith 2023).

### Screening, preparation and photography

The amber was observed and initially screened for inclusions with a light microscope using both transmitted and incident light. All insect remains and impressions were found in the amber from Orp-le-Grand. As the amber is extremely brittle and the hemipteran inclusion was exposed very close to the surface of the amber with a fissure exposing part of the specimen, the amber specimen was stabilized by applying a very thin coat of high-grade epoxy resin (Epo-Tek 301-2, Epoxy Technology, Billerica, Massachusetts, USA), that also sealed the existing fissures (see Sadowski et al.^59^, for protocols), before minor and careful grinding and polishing of the specimen. Grinding and polishing manually with wet silicon carbide papers (grit from 25.8 to 5.0 μm particle size; Struers company, USA) was done to remove scratches and to create a smooth surface parallel to the inclusion.

The amber inclusions were examined with a Stereo Discovery V8 dissection microscope (Carl Zeiss, Germany) and an AxioScope A1 compound microscope (Carl Zeiss, Germany) using incident and transmitted light simultaneously. Images were taken with Canon EOS 5D digital cameras (Canon, Tokyo, Japan) attached to these microscopes. Additional photographs were taken at the Laboratory of Evolutionary Entomology and Museum of Amber Inclusions at the University of Gdansk, with an Olympus BX51 microscope (Olympus, Tokyo, Japan), equipped with a Canon EOS 90D digital camera (Cannon Tokyo, Japan) abd ab Oympus EP50 camera. The images were then stacked using HeliconFocus version 6.3.3 Pro (Helicon Soft, Kharkov, Ukraine). Some images are composites of several stacked images stitched together using Adobe Photoshop. For the insect wing impressions, the images were taken using a Stemi 508 dissecting microscope (Carl Zeiss, Germany) with incident light and the images stacked using Photoshop. Drawings were made with the use of camera lucida attached to the Olympus BX51 and Olympus SZX10 microscopes then readjusted with the CorelDrawX7 package (Corel, Ottawa, Canada).

### FTIR and statistical analyses

Samples of the Belgium ambers (three per locality were allowed by RBINS, Supplementary Fig. 1) along with comparison samples of Oise amber deriving from the Le Quesnoy quarry (Paris Basin) (three samples measured, Supplementary Fig. 1), plus samples of Baltic succinite and Burmese amber (Supplementary Fig. 2), were finely powdered separately prior to measurement. The spectra were collected as detailed in Seyfullah et al.^60^, using attenuated total reflection (ATR) mode with a Bruker Platinum A225 diamond ATR accessory installed on an FTIR spectrometer (Bruker Vertex 70), in absorbance with 32 scans collected for each sample with a spectral resolution of 4 cm^−1^ in the range from 4000 to 650 cm^−1^. Background spectra were taken before each analytical run and automatically used to correct for background from the sample spectra. Each sample was measured three times to ensure consistency and three samples of the different ambers were used in total. Bands were identified by comparison with previous reports^26–29^. The ATR crystal and anvil were thoroughly cleaned between each measurement. The spectra were visualized using SpectraGryph v1.2.13. The spectra were then subjected to statistical analyses.

All data manipulation and analysis were carried out in R version 4.1.0^61^ with the packages baseline version 1.3–1^62^ and RColorBrewer version 1.1–2^63^. The dataset and R script are available in the supplementary information. Each spectrum was standardized to zero mean and unit variance (z-scores) using the equation (x−x̄)/σ, where x is the absorbance value, x̄ is the spectrum arithmetic mean, and σ is the spectrum standard deviation. The baseline was corrected using a modified polynomial fitting with a second order polynomial baseline^61^.

For the multivariate data exploration and visualization principal component analysis (PCA) and cluster analysis were used^64^. The cluster analysis was run using the Euclidean distance and with the unweighted pair group method with arithmetic mean (UPGMA) linkage algorithm and the locality-mean spectra were also subject to this to simplify relationships between the amber samples from the different localities. Other clustering options were also run. The datasets and the R scripts are available as Supplementary data.

### Terminology and taxonomy

This publication and the nomenclatural acts it contains are registered in ZooBank, the proposed online registration system for the International Code of Zoological Nomenclature (ICZN). The ZooBank LSIDs (Life Science Identifiers) can be obtained and the associated information viewed using any standard web browser by appending LSID to the prefix ‘http://zoobank.org/’. The LSID for this publication is: urn:lsid:zoobank.org:pub:BB11FBB1-5FEC-4994-A8B2-0DAD2D082B70.

### Supplementary Information

Supplementary Information 1.

Supplementary Information 2.

Supplementary Information 3.

Supplementary Information 4.

Supplementary Information 5.

Supplementary Figures.

## Data Availability

All data and R codes generated and analysed during this study are supplied as files for download and so are included in this published article and Supplementary Figs. 1–4 are available as a pdf download for this publication.
