# Supplementary material for: Chemical and palaeoentomological evidence of a relationship between early Eocene Belgian and Oise (France) ambers
Source: Sci Rep. 2024 Jun 14;14:13705. doi: 10.1038/s41598-024-64286-z (PMC11176311; doi:10.1038/s41598-024-64286-z)
Supplement: Supplementary file 6 — Supplementary Figures. [file 41598_2024_64286_MOESM6_ESM.pdf]

Chemical and palaeoentomological evidence of a relationship between early Eocene Belgian and Oise (France) ambers

Leyla J. Seyfullah,[1*#], Jacek Szwedo[2#], Alexander R. Schmidt[3] & Cyrille Prestianni[4,5]

Supplementary Information

SI Dataset and R scripts (separate Zip file) and five SI figures

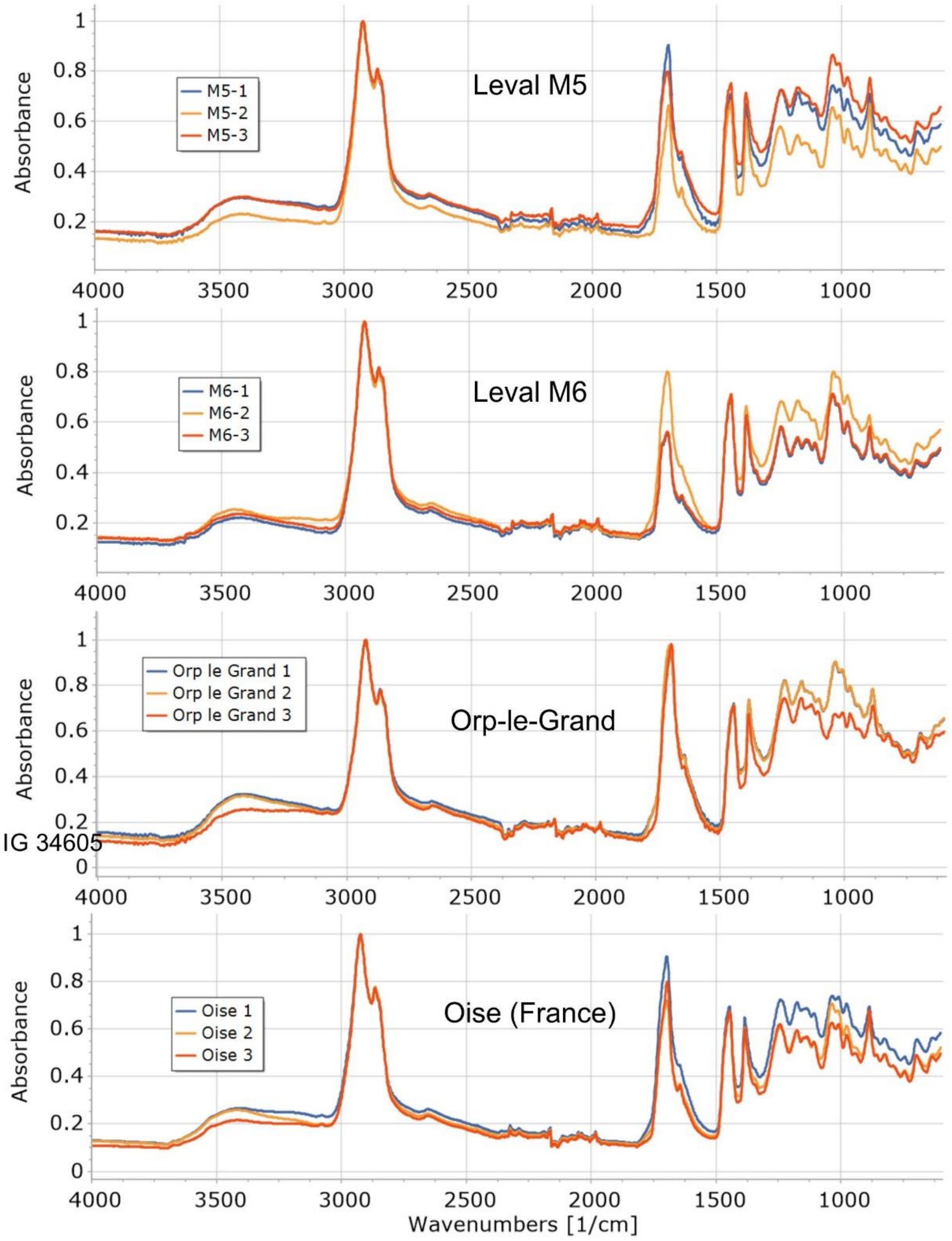

Figure S1. FTIR Spectra for each sample of Eocene amber from each locality. Belgian localities: Leval M5, Leval M6 and Orp-le-Grand. Oise amber derives from the Le Quesnoy quarry, Paris Basin, France.

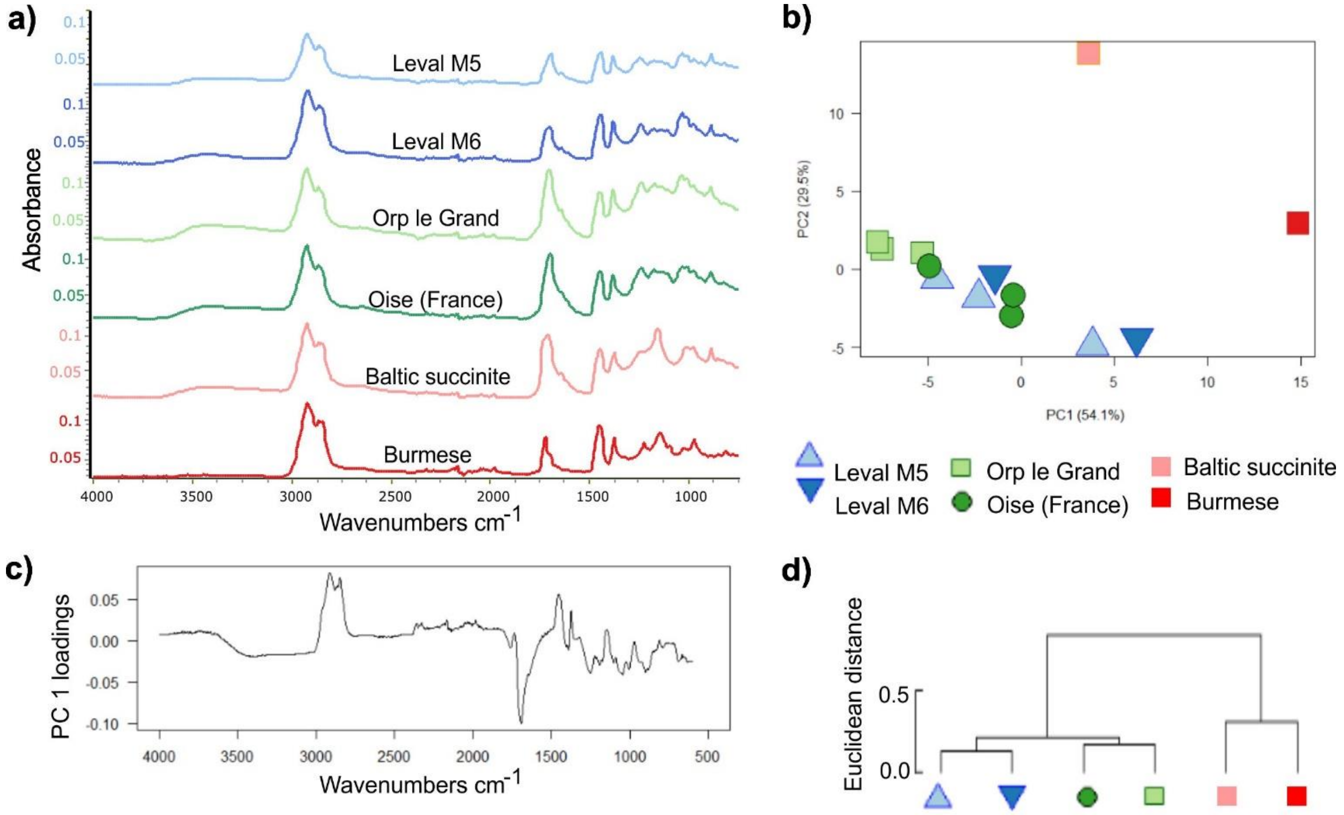

Figure S2. Analyses of Belgian and Oise ambers with Baltic succinite and Burmese amber FTIR spectra added. a) Mean FTIR spectra by locality for the Eocene ambers from Belgium (Leval M5, Leval M6 and Orp-le-Grand), Oise (France), Baltic succinite and Burmese amber. b)-d) Multivariate analysis of amber spectra from Belgium (Leval M5, Leval M6 and Orp-le-Grand), Oise (France), Baltic succinite and Burmese amber using FTIR-ATR spectroscopy: b) Principal components analysis showing PC1 vs. PC2, note the two triangles for Leval M6 – the more central one is composed of two triangles, one superimposed on the other, giving the appearance that one sample is missing when in fact two are indistinguishable; c) loadings for PC1; d) mean cluster analysis by locality.

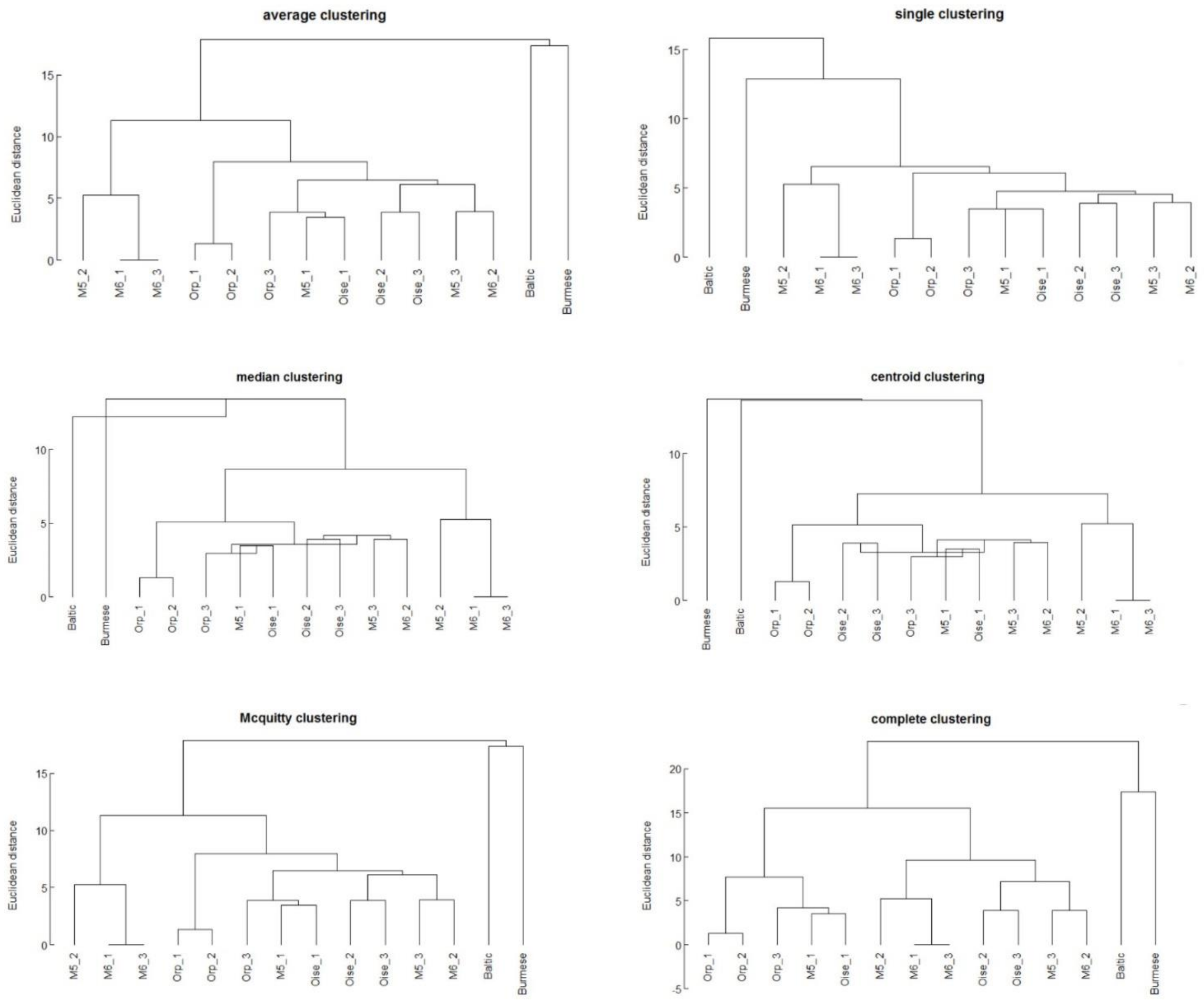

Figure S3. Different clustering methods applied to the individual amber spectra.

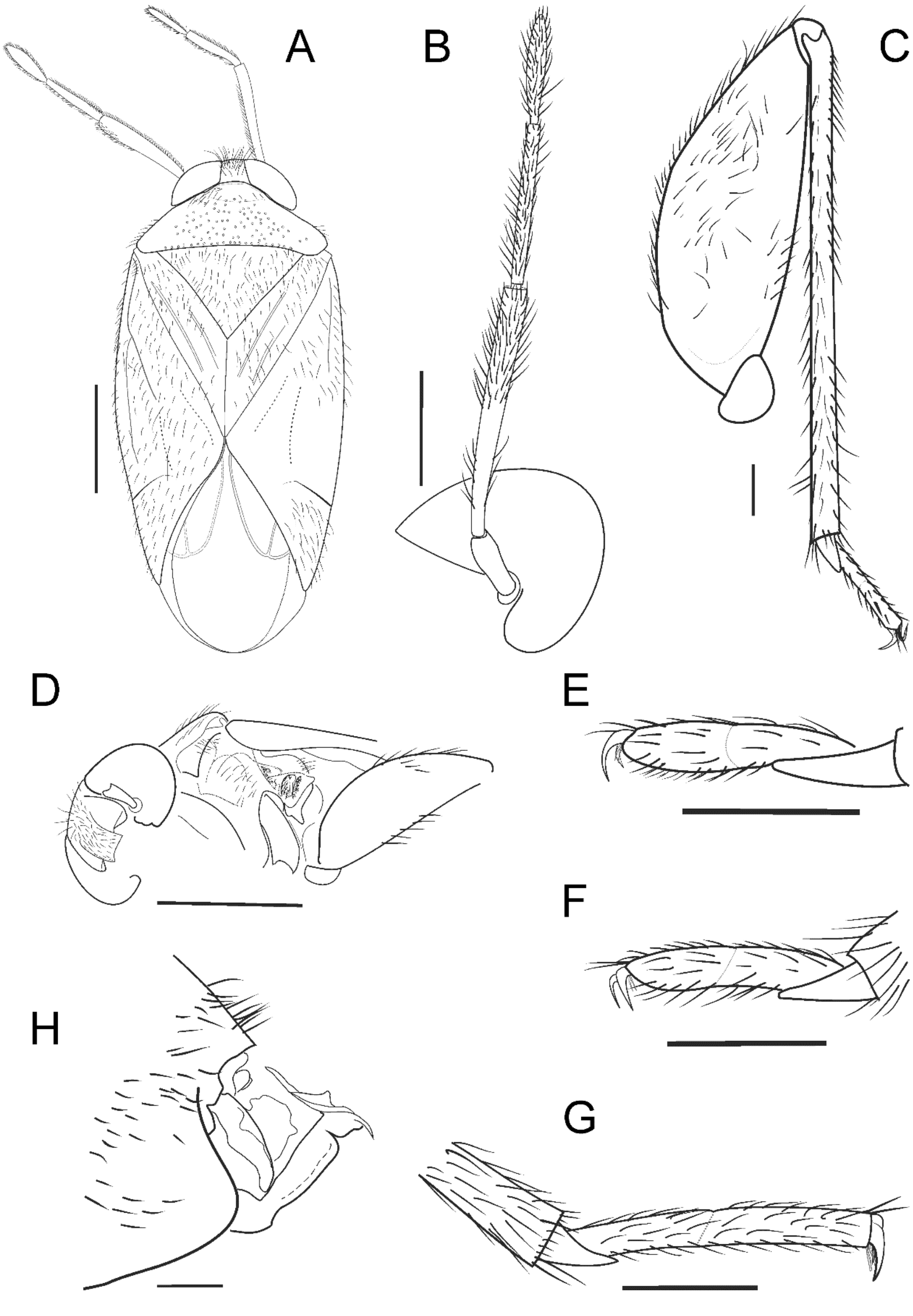

Figure S4. *Cativolcus uebruum* gen. et sp. nov. A. Body general view; B. Antenna; C. Metaleg; D. Thorax in ventrolateral view; E. Protarsus; F. Mesotarsus; G. Metatarsus; H. Male genital block. Scale bars: 0.5 mm for A and D, 0.1 mm for B, C, E-H.

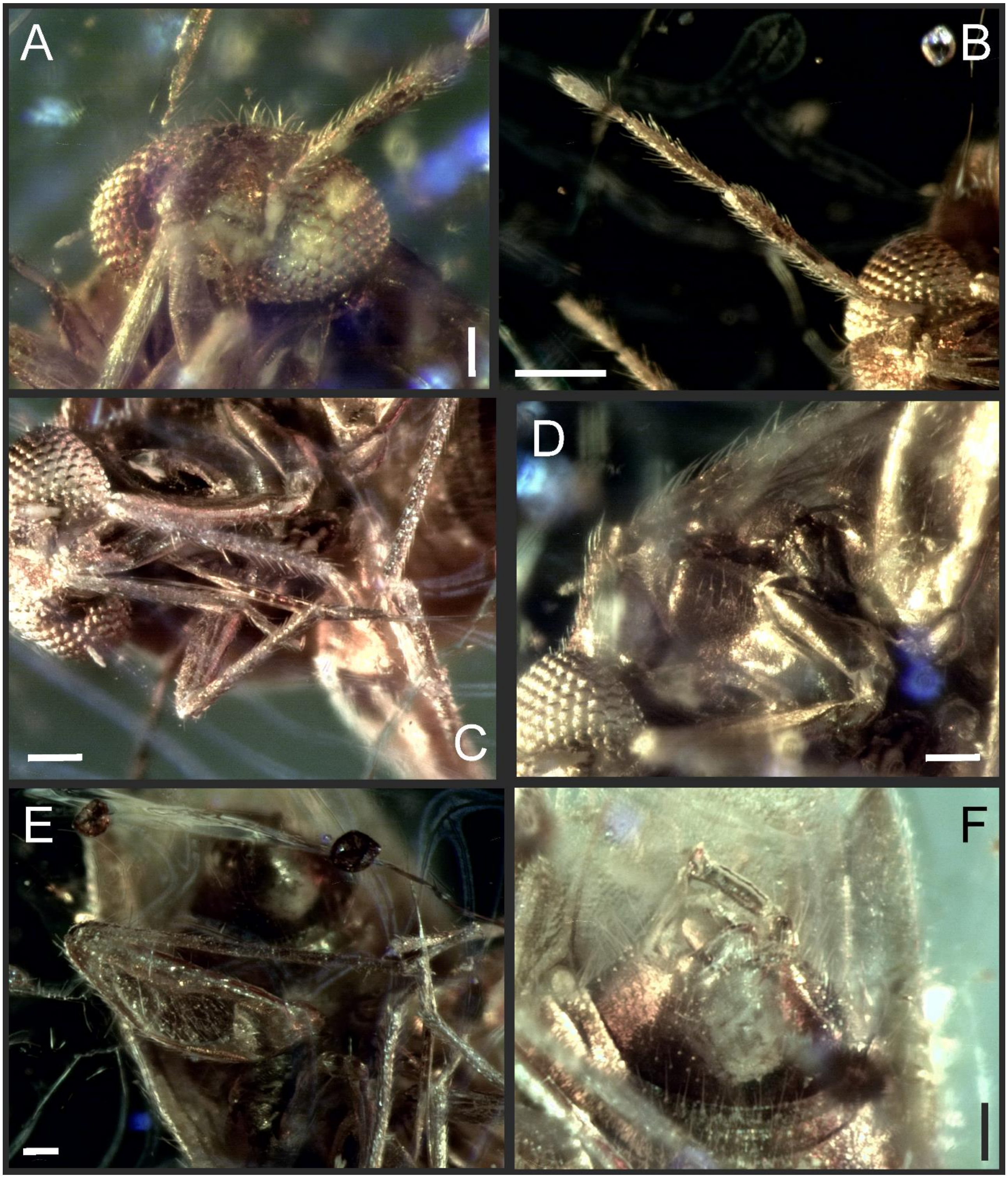

Figure S5. *Cativolcus uebruum* gen. et sp. nov. A. Face; B. Antenna; C. Rostrum and prolegs, ventral view; D Thorax, ventrolateral view; E. Metaleg; F. Male genital block. Scale bars: 0.1 mm for A, C-F, 0.2 mm for B.
